# Statistical Inference in Hidden Markov Models Using *k*-Segment Constraints

**DOI:** 10.1080/01621459.2014.998762

**Published:** 2016-05-05

**Authors:** Michalis K. Titsias, Christopher C. Holmes, Christopher Yau

**Keywords:** Dynamic programming, Hidden Markov models, Segmentation.

## Abstract

Hidden Markov models (HMMs) are one of the most widely used statistical methods for analyzing sequence data. However, the reporting of output from HMMs has largely been restricted to the presentation of the most-probable (MAP) hidden state sequence, found via the Viterbi algorithm, or the sequence of most probable marginals using the forward–backward algorithm. In this article, we expand the amount of information we could obtain from the posterior distribution of an HMM by introducing linear-time dynamic programming recursions that, conditional on a user-specified constraint in the number of segments, allow us to (i) find MAP sequences, (ii) compute posterior probabilities, and (iii) simulate sample paths. We collectively call these recursions *k*-segment algorithms and illustrate their utility using simulated and real examples. We also highlight the prospective and retrospective use of *k*-segment constraints for fitting HMMs or exploring existing model fits. Supplementary materials for this article are available online.

## INTRODUCTION

The use of the hidden Markov model (HMM) is ubiquitous in sequence analysis applications across a range of science and engineering domains, including signal processing (Crouse, Nowak, and Baraniuk 1998), genomics (Li and Stephens 2003), and finance (Paas, Vermunt, and Bijmolt 2007). The HMM is a mixture model whose mixing distribution is a finite state Markov chain (Rabiner 1989). While Markov assumptions rarely correspond to the true physical generative process, they often adequately capture dependencies that allow the HMM to be a useful approximating model that is tractable even for very large datasets. As a consequence, HMM-based algorithms can give highly competitive performance in many applications.

Central to the tractability of HMMs is the availability of recursive algorithms that allow fundamental quantities to be computed efficiently (Baum and Petrie 1966; Viterbi 1967). These include the Viterbi algorithm that computes the most probable hidden state sequence and the forward–backward algorithm that computes the marginal probability of a given state at a point in the sequence. Computation for the HMM has been well summarized in the comprehensive and widely read tutorial by Rabiner (1989) with a Bayesian treatment given more recently by Scott (2002). It is a testament to the completeness of these recursive methods that there have been few generic additions to the HMM toolbox since these were first described in the 1960s. However, as HMM approaches continue to be applied to increasingly diverse scientific domains and ever larger datasets, there is interest in expanding the generic toolbox available for HMM inference to encompass unmet needs, particularly in hypothesis generation for scientific discovery-driven applications.

The motivation for our work is to develop mechanisms that will be used to *explore* larger subsets of sequences that may be of application-specific utility. Typically, standard HMM inference limits itself to reporting a few standard quantities. For an *M*-state Markov chain of length *N*, there exists *M^N^* possible sequences but often only the most probable sequence or the *NM* marginal posterior probabilities are used to summarize the whole posterior distribution. Yet, it is clear that, when the state space is large and/or the sequences are long, many other statistics maybe of interest. Modifications of the Viterbi algorithm can allow arbitrary numbers of the most probable sequences to be enumerated while Bayesian techniques allow us to sample sequences from the posterior distribution. However, since a small change to the most likely sequences typically give new sequences with similar probability, these approaches do not lead to reports of *qualitatively diverse* sequences. By which we mean, alternative sequence predictions that might lead to different decisions or scientific insights. This can be particularly important where the sequence analysis forms only part of an iterative investigative process where the users might later return to the data to explore additional features.

In this article, we describe a set of novel recursive methods for HMM computation that incorporates segmental constraints that we call *k*-*segment inference algorithms*. These algorithms are constrained to consider only sequences with a prespecified number of transition events allowing diverse sequence predictions to be obtained. Further, these methods can be applied prospectively during model fitting or retrospectively to an existing model. In the latter case, the utility of the methods described here comes at no cost (other than computational time) to the HMM user.

## 2. MOTIVATION

Our work is motivated by two real world applications in genomics and information retrieval. The first concerns the use of whole genome microarray or sequence analysis for the identification of DNA copy number alterations. The objective of DNA copy number analysis is to segment the observed sequence coverage signal into homogenous regions of constant signal intensity and then to classify these segments in terms of their DNA copy number. A popular class of methods uses HMMs for this purpose where the observed sequence read counts are used to infer a sequence of latent copy number states (Greenman et al. 2010; Yau et al. 2010; Chen, Xing, and Zhang 2011; Li et al. 2011).

**Figure 1:**
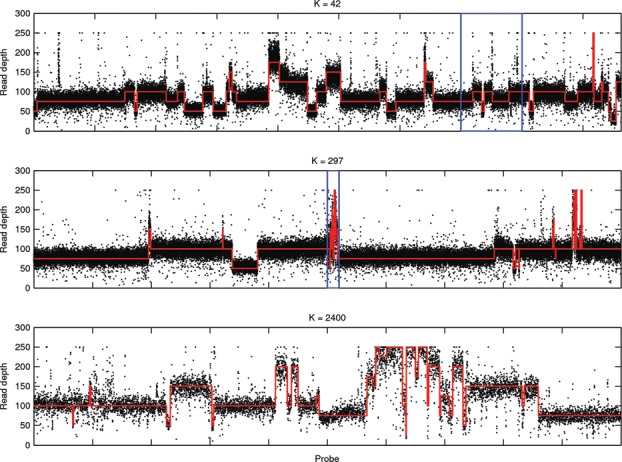
Whole genome DNA copy number analysis. Segmentation of the observed sequencing read depth along the genome can be used to identify changes in the underlying DNA copy number. (Top) Broad level changes can be adequately captured using a relatively small number of segments but if we zoom in on the labeled region (blue) higher resolution segmentations (middle/bottom) can require thousands of segments.

Figure 1 shows genome-wide sequence coverage for a genomically unstable colorectal cancer harboring complex DNA copy number changes. Broad level copy number changes in the genome can be characterized by a segmentation requiring only 48 segments, but hundreds to thousands of segments may be required to capture finer scale details. Ordinarily, methods implicitly target the high-resolution objective but these results can be unwieldy and difficult to use. Low-resolution alternatives may offer sufficient detail for qualitative description and subsequent scientific investigation. In practice, low-resolution summaries are often obtained from high-resolution segmentations by using post-processing heuristics to merge segments. We will demonstrate that our *k*-segment methods provide a more principled approach for accessing segmentations with a range of complexities that can be applied retrospectively to existing HMM implementations.

In our second example, we will examine an information retrieval example where the objective is to analyze text documents and to determine if they contain phrases belonging to certain topics. Here, we will show the utility of *k*-segment algorithms for counting occurrences of topic segments in textual documents and to evaluate inequalities, in this case, the probability that there is *at least* one phrase corresponding to a certain topic. We show that decision systems based upon such measures rather than point estimates (the Viterbi sequence) lead to more robust classification performance.

Overall, the *k*-segment algorithms we present are naturally useful in scientific discovery problems involving (i) the application of HMMs and (ii) where segmental constraints provide an important source of external information or constraints. Our methods can be used to guide the selection of sequence predictions for follow-up investigation and validation.

## 3. BACKGROUND

The HMM encodes for two types of random sequences: the hidden state sequence or path $x=(x_{1},...,x_{N})$ and the observed data sequence $y=(y_{1},...,y_{N})$. Individual hidden states take discrete values, such that *x_n_* ∈ {1, …, *M*}, while observed variables can be of arbitrary type. The hidden state sequence x follows a Markov chain so that

(1) $$p\left(x|\pi_{0},A\right)=p\left(x_{1}|\pi_{0}\right)\prod_{n=2}^{N}p\left(x_{n}|x_{n-1},A\right).$$

Here, the first hidden state *x*_1_ is drawn from some initial probability vector $\pi_{0}$ so that π_0, *m*_ = *p*(*x*_1_ = *m*) denotes the probability of *x*_1_ being in state *m* ∈ {1, …, *M*}, whereas any subsequent hidden state *x_n_* (with *n* > 1) is drawn according to a transition matrix *A* so that ${\left[A\right]}_{m^{'}m}=p\left(x_{n}=m|x_{n-1}=m^{'}\right)$ expresses the probability of moving to a state *m* from *m*′. Given a path x following the Markov chain in (1), the observed data are generated independently according to

(2) $$p\left(y|x\right)=\prod_{n=1}^{N}p\left(y_{n}|x_{n},\varphi \right),$$

where the densities *p*(*y_n_*|*x_n_* = *m*, φ), *m* = 1, …, *M*, are often referred to as the emission densities and are parameterized by φ. In what follows we shall collectively denote all HMM parameters, that is, $\pi_{0}$, *A*, and φ, by θ.

Statistical estimation in HMMs takes advantage of the Markov dependence structure that allows efficient dynamic programming algorithms to be applied. For instance, maximum likelihood (ML) over the parameters θ via the EM algorithm is carried out by the forward–backward (F-B) recursion (Baum and Petrie 1966) that implements the expectation step in *O*(*M* ^2^ *N*) time. A similar recursion having the same time complexity is the Viterbi algorithm (Viterbi 1967) which, given a fixed value for the parameters, estimates the maximum a posteriori (MAP) hidden sequence. Furthermore, straightforward generalizations of the Viterbi algorithm estimate the *P*-best list of most probable sequences (Schwartz and Chow 1990; Nilsson and Goldberger 2001). In contrast to ML point estimation, a Bayesian approach assigns a prior distribution p(θ) over the parameters and seeks to estimate expectations taken under the posterior distribution p(x,θ|y). The Bayesian framework also greatly benefits from efficient recursions derived as subroutines of Monte Carlo algorithms. Specifically, the popular Gibbs sampling scheme (Scott 2002) relies on the forward-filtering-backward-sampling (FF-BS) recursion that simulates in *O*(*M* ^2^ *N*) time a hidden sequence from the conditional posterior distribution p(x|θ,y). In summary, all recursions mentioned above have linear time complexity with respect to the length of the sequence *N* and are instances of more general inference tools developed in the theory of probabilistic graphical models (Cowell et al. 2003; Koller and Friedman 2009).

## 4. THEORY OF *k*-SEGMENT INFERENCE

We now present the theoretical foundations of *k*-segment inference. The methods described in this section assume a fixed setting for the parameters θ. Therefore, to keep our expressions uncluttered in the following we drop θ from our expressions and write for instance p(x|y,θ) as p(x|y) and p(y|θ) as p(y).

### 4.1 *k*-Segment Inference Problems

Any hidden path x in an HMM can have from 0 up to *N* − 1 transitions or equivalently from 1 up to *N* segments, where a segment is defined as a contiguous run of indices where *x*_*n* − 1_ = *x_n_*. We define the number of all segments in x by

(3) $$c_{x}=1+\sum_{n=2}^{N}I\left(x_{n-1}\neq x_{n}\right),$$

where *I*( · ) denotes the indicator function. *c_x_* is the sum of the number of transitions, that is, the locations in the hidden path where *x*_*n* − 1_ ≠ *x_n_*, and the value 1 that accounts for the initial segment, which is not the result of a transition.

Subsets of hidden paths associated with different number of segments comprise exclusive events that allow to decompose the posterior distribution p(x|y) as follows. If we introduce the events *c_x_* = *k*, with *k* = 1, …, *N*, each corresponding to the subset of paths $\left\{x|c_{x}=k\right\}$ having exactly *k* segments, the posterior distribution p(x|y) can be written as the following mixture:

(4) $$p\left(x|y\right)=\sum_{k=1}^{N}p\left(x,c_{x}=k|y\right)=\sum_{k=1}^{N}p\left(x|c_{x}=k,y\right)p\left(c_{x}=k|y\right),$$

where

(5) $$p\left(x|y,c_{x}=k\right)=\frac{I\left(c_{x}=k\right)p\left(y|x\right)p\left(x\right)}{\sum_{x:c_{x}=k}p\left(y|x\right)p\left(x\right)}$$

is the posterior distribution conditional on having *k* segments, while

(6) $$p\left(c_{x}=k|y\right)=\frac{p(c_{x}=k,y)}{p(y)}=\frac{\sum_{x:c_{x}=k}p\left(y|x\right)p\left(x\right)}{\sum_{x}p\left(y|x\right)p\left(x\right)}$$

is the posterior probability of the event *c_x_* = *k*.

The mixture decomposition in Equation (4) suggests that one way to explore the posterior distribution of the HMM is to compute quantities associated with the components of this mixture. This leads to the *k*-segment inference problems that can be divided into the following three types of problems:

- *Optimal decoding:* Find the MAP hidden path that has *k* segments, that is, the path with the maximum value of $p(x|c_{x}=k,y)$;
- *Probability computation:* Find the posterior probability of having *k* segments, that is, $p(c_{x}=k|y)$; and
- *Path sampling:* Draw independent samples from $p(x|c_{x}=k,y)$.

To this end, we introduce efficient linear time algorithms to solve all the above tasks together with several additional related tasks associated with more general events of the form *k*_1_ ⩽ *c_x_* ⩽ *k*_2_, where 1 ⩽ *k*_1_ < *k*_2_ ⩽ *N*, such as finding the MAP of $p(x|c_{x}>k,y)$, sampling from $p(x|c_{x}>k,y)$, etc. These algorithms are based on a reformulation of the above *k*-segment inference problems that uses an extended state-space HMM containing auxiliary counting variables.

### 4.2 Auxiliary Counting Markov Chains

The basis of our algorithm is the augmentation of the Markov chain in (1) with auxiliary variables that count the number of segments. Specifically, the general count *c_x_* from (3) can be considered as a counter that scans the path x and it increments by one any time it encounters a transition. We can represent this counting process with an *N*-dimensional vector of auxiliary variables s, which is an increasingly monotone sequence of nonnegative integers, that is, $s_{n}=c_{x_{1:n}}$.

Conditioning on a certain path x, s is sampled deterministically according to the Markov chain

(7) $$\begin{array}{ccc} p(s|x) & = & p\left(s_{1}|x_{1}\right)\prod_{n=2}^{N}p\left(s_{n}|s_{n-1},x_{n-1},x_{n}\right),\\ & = & \delta_{s_{1},1}\prod_{n=2}^{N}\left[I\left(x_{n-1}\neq x_{n}\right)\delta_{s_{n},s_{n-1}+1}\right.\\ & & +\left.\left(1-I\left(x_{n-1}\neq x_{n}\right)\right)\delta_{s_{n},s_{n-1}}\right], \end{array}$$

where δ_*i*, *j*_ is the delta mass that equals one when *i* = *j* and zero otherwise. We refer to the above conditional distribution as the *counting Markov chain* or counting chain because it is Markov chain that makes precise the concept of counting the segments. The counting chain starts at one, that is, *s*_1_ = 1 (which can be interpreted as sampling from the delta mass $\delta_{s_{1},1}$), and then it increments by one so that *s_n_* = *s*_*n* − 1_ + 1 every time a transition occurs in the hidden path, that is, whenever *x*_*n* − 1_ ≠ *x_n_*, which implies the generation of a new segment. The joint density of the HMM is augmented with the counting chain so that

(8) p(y,x,s)=p(y|x)p(x)p(s|x).

As the augmentation leaves the joint distribution between y and x unaltered (if we marginalize out s, we recover correctly the joint density of the initial HMM), prior-to-posterior inference in the initial HMM and the HMM augmented with auxiliary variables are equivalent. However, in practice, inference in the latter model is more flexible since it allows us to solve the *k*-segment inference problems through the insertion of constraints in the counting process. More precisely, given that the final value of the counter *s_N_* equals *c_x_*, all type of *k*-segment inference problems can be reformulated as follows:

- *Optimal decoding:* The MAP hidden $x^{*}$ of $p(x|c_{x}=k,y)$ can be found according to
  (9) $$\left(x^{*},s_{\setminus N}^{*}\right)=\arg\;\max_{x,s_{\setminus N}}p\left(y|x\right)p\left(x\right)p\left(s_{\setminus N},s_{N}=k|x\right),$$
  where in the above $s_{\setminus N}$ denotes all counting variables apart from the final *s_N_*, which is clamped to *k*.
- *Probability computation:* The posterior probability $p(c_{x}=k|y)$ can be expressed as $\frac{p(s_{N}=k,y)}{p(y)}$, where p(y) is known from the forward pass of the standard F-B algorithm and
  (10) $$p\left(s_{N}=k,y\right)=\sum_{x,s_{\setminus N}}p\left(y|x\right)p\left(x\right)p\left(s_{\setminus N},s_{N}=k|x\right).$$
- *Path sampling:* An independent sample $\tilde{x}$ from $p(x|c_{x}=k,y)$ is obtained as
  (11) $$\begin{array}{ccc} & & \left(\tilde{x},{\tilde{s}}_{\setminus N}\right)∼p\left(x,s_{\setminus N}|s_{N}=k,y\right)\propto p\left(y|x\right)p\left(x\right)\\ & & \;\times p(s_{\setminus N},s_{N}=k|x). \end{array}$$

For more general events of the form *k*_1_ ⩽ *s_N_* ⩽ *k*_2_, where 1 ⩽ *k*_1_ < *k*_2_ ⩽ *N*, the above still holds with the slight modification that we will need additionally to maximize, marginalize, or sample *s_N_*, respectively, for the three cases above, under the constraint *k*_1_ ⩽ *s_N_* ⩽ *k*_2_. Simple proofs for the correctness of all above statements can be found in supplementary materials.

Furthermore, the *k*-segment inference problems associated with the special case of the event *s_N_* > *k* can be equivalently reformulated by using a modified counting chain that absorbs when *s_n_* = *k* + 1, that is,

(12) $$\begin{array}{ccc} p(s|x) & = & \delta_{s_{1},1}\prod_{n=2}^{N}\left[I\left(x_{n}\neq x_{n-1}\;\&\;s_{n-1}\leq k\right)\delta_{s_{n},s_{n-1}+1}\right.\\ & & +\left.\left(1-I(x_{n}\neq x_{n-1}\;\&\;s_{n-1}\leq k)\right)\delta_{s_{n},s_{n-1}}\right], \end{array}$$

where the indicator function $I(x_{n}\neq x_{n-1}\;\&\;s_{n-1}\leq k)$ is one only when both *x_n_* ≠ *x*_*n* − 1_ and *s*_*n* − 1_ ⩽ *k* are true. Notice that the above is an inhomogenous chain having two modes: the first when the segment counting proceeds normally and the second when counting stops once the absorbing state is visited. The *k*-segment problems for the event *s_N_* > *k* are then solved by using the above chain and clamping *s_N_* to the value *k* + 1.

The augmentation with counting variables results in a new HMM having the pair (*s_n_*, *x_n_*) as the new extended state variable. Given that *s_N_* = *k*, so that any pair (*s_n_*, *x_n_*) can jointly take at most *kM* values, we can use the Viterbi algorithm to obtain the MAP of $p(x|y,s_{N}=k)$, the forward pass of the F-B algorithm to obtain $p(s_{N}=k,y)$ and the FF-BS algorithm to draw an independent sample from $p(x|y,s_{N}=k)$. A naive implementation of these algorithms can be done in *O*(*k* ^2^ *M* ^2^ *N*) time but this complexity can be further reduced to *O*(*kM* ^2^ *N*) by taking into account the deterministic structure of the counting chain using dynamic programming-based algorithms. Furthermore, the dynamic programming algorithms can solve at once the corresponding *k*-segment inference problems from *k* = 1 up to a maximum *k* = *k*_max_ in overall *O*(*k*_max_ *M* ^2^ *N*) operations. Also, by running the *k*-segment Viterbi algorithm up to some *k*_max_ and setting *k*_max_ + 1 as the absorbing counting state it always gives a global summary of the posterior distribution, consisting of *k*_max_ + 1 optimal paths associated with the events *c_x_* = 1, …, *c_x_* = *k*_max_ and *c_x_* > *k*_max_ that is guaranteed to include the standard Viterbi MAP path. Such a summary is referred to as *k*_max_ + 1 summary and it is illustrated in the next section. Further details regarding the implementation of the dynamic programming methods are discussed in supplementary materials.

## 5. COMPARING *k*-SEGMENT AND STANDARD HMM RECURSIONS

In this section, we discuss two established HMM recursions for extracting summaries and a comparison of their performance with *k*-segments. These include the FF-BS algorithm for simulating exact paths from the posterior p(x|y) or the best list Viterbi (BL-Viterbi) algorithm (Schwartz and Chow 1990) that extracts a set of paths having the highest posterior probability. We demonstrate that while both approaches report highly probable sequences, this does not lead to reporting diverse summaries.

For this, we simulated a data sequence according to $y_{n}|x_{n},m,\sigma^{2}∼N\left(m_{x_{n}},\sigma^{2}\right),n=1,\cdots ,N=1000$, where the hidden sequence $x={\left\{x_{n}\right\}}_{n=1}^{N}$ was given by a Markov chain with *M* = 3 states, m={-2,-1,1}, and σ = 0.9. The transition matrix used was

$$\begin{array}{c} A=\left(\begin{array}{ccc} 0.98 & 0.015 & 0.005\\ 0.005 & 0.98 & 0.015\\ 0.015 & 0.005 & 0.98 \end{array}\right) \end{array}$$

while the prior distribution was uniform.

**Figure 2:**
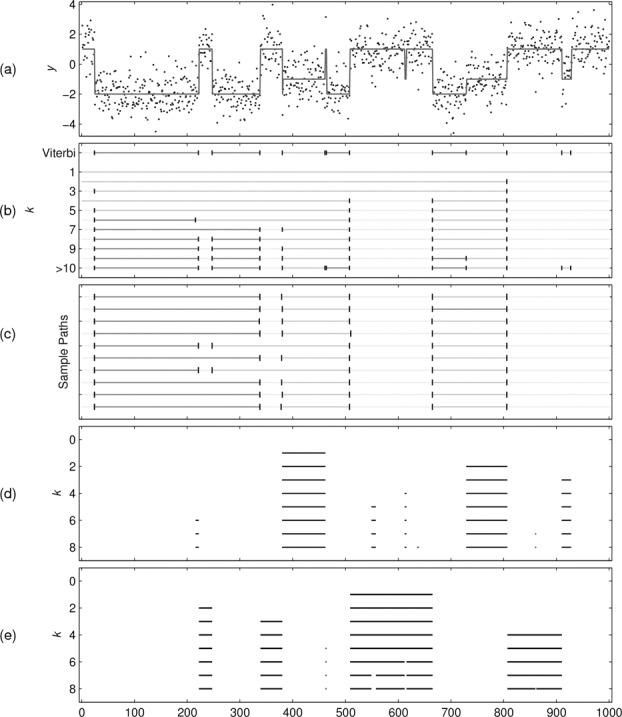
(a) Simulated data sequence. (b) Viterbi segmentation and *k* = 1…10, >10 paths from *k*-segment inference. (c) 10 sample paths obtained by the FF-BS algorithm under the constraint *k* = 7. (d) Paths with 0–8 segments from State 2 obtained using generalized counting constraints. (e) Counting excursions from null (State 1/2) to abnormal (State 3) states. States 1, 2, and 3 have mean levels −2, −1, and 1, respectively.

Using the simulated data, we fitted a three-state HMM using the EM algorithm that recovered parameter estimates close to the true values used in the simulation. We then computed the standard Viterbi path (containing 14 segments) and obtained the optimal segmentations using *k*-segments for *k*_max_ = 10, including the *k*_max_ + 1 summary. These are shown in Figure 2.

The first 10 paths of the *k*-segments summary provide a coarse-to-fine hierarchical segmentation of the data sequence where the number of segments increases by one each time. Notice that two consecutive segmentations do not always follow the principle used in the circular binary segmentation algorithm (Olshen et al. 2004), that is, the *k* + 1th segmentation might not be obtained by splitting into two segments a single segment from the *k*th one. This latter approach is suboptimal. Also, notice that the final path that corresponds to the absorbing state (labeled with > 10 in the figure) is precisely the standard Viterbi path. Figure 2 also illustrates path sampling under *k*-segment constraints using the FF-BS algorithm in the augmented HMM. In particular, 10 samples are shown that are constrained to have exactly *k* = 7 segments.

**Figure 3:**
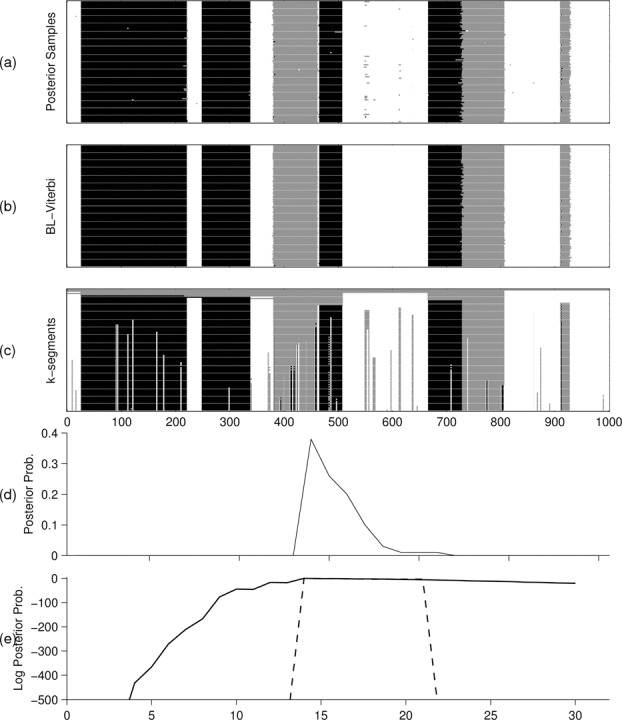
Comparison of *k*-segment paths with standard summaries. (a) 100 posterior samples obtained by FF-BS, (b) 100 most probable paths obtained by BL-Viterbi, (c) *k* = 1, …, 100 paths obtained by *k*-segments, (d) posterior distribution over segment number, and (e) log-posterior distribution obtained by *k*-segments (-) and by Monte Carlo (- -) using FF-BS.

We investigated whether the FF-BS and BL-Viterbi algorithms could provide posterior summaries that showed diversity in terms of the number of segments in the reported paths. We applied the FF-BS recursion to collect 100 independent samples from p(x|y) and used the BL-Viterbi algorithm to extract the top 100 paths having the highest posterior probability. Figure 3 shows that these paths exhibit limited diversity and there was no path having less than 14 segments. Most of these paths are minor perturbations of one another typically at the boundaries between segments. Paths with very small but, nonzero, posterior probabilities (less than 14 segments) are very unlikely to be realized in practice. In contrast, the *k*-segment recursion guarantees to provide different segmentations of the observed sequence.

Similarly, the use of the standard FF-BS recursion as a means of providing a Monte Carlo approximation of the segment number probability $p(c_{x}=k|y)$ is also unsuitable when the true value of $p(c_{x}=k|y)$ is very small. Figure 3 shows the Monte Carlo estimates of the (log) posterior probabilities obtained from 1000 independent samples. This differs significantly from the corresponding exact probabilities computed via *k*-segments. Exact probability computation would be useful in decision theoretical framework where we wish to build decision-making systems that involve utility functions that favor extreme events.

## 6. LEARNING WITH *k*-SEGMENT CONSTRAINTS

So far we have presented novel recursions for HMM inference that are applied retrospectively to a fitted HMM. In this section, we discuss how we could use these recursions in a *prospective* statistical estimation problem with HMMs where the constraints are introduced during model fitting so that they actively influence the inference for model parameters. We consider both point estimation using the expectation–maximization (EM) algorithm and posterior sampling in a Bayesian context.

### 6.1 Expectation–Maximization

Consider the joint density of the augmented HMM:

(13) $$p\left(y,x,s\right)=p\left(y|x\right)p\left(x\right)p\left(s_{N}\leq k,s_{\setminus N}|x\right),$$

where the evidence *s_N_* ⩽ *k* reflects the information about the maximum number of segments allowed.

We would like now to apply the EM algorithm to learn the parameters θ for which we need to write down the auxiliary *Q* function and subsequently derive the E and M steps:

(14) $$Q\left(\theta ;\theta^{\text{old}}\right)=E_{p(x|s_{N}\leq k,y,\theta^{\text{old}})}\left[\log p\left(y|x,\theta \right)p\left(x,\theta \right)\right]+\text{const},$$

where $\theta^{\text{old}}$ denotes the current parameter values. This function has exactly the same form with the auxiliary function in the unconstrained HMM with the only difference being that $p(x|y,\theta^{\text{old}})$ is replaced by $p(x|s_{N}\leq k,y,\theta^{\text{old}})$.

The E step simplifies to computing all marginals $p(x_{n}|s_{N}\leq k,y,\theta^{\text{old}})$ and all pairwise marginals $p(x_{n-1},x_{n}|s_{N}\leq k,y,\theta^{\text{old}})$, which can be obtained by applying the F-B algorithm in the augmented HMM. Given the current $\theta^{\text{old}}$ (omitted next for brevity), this algorithm computes the forward (α) messages and the backward (β) messages (for details see supplementary materials) from which the desired marginals and pair-wise marginals can be obtained

(15) $$\begin{array}{ccc} & & p\left(x_{n}|s_{N}\leq k,y\right)\propto \sum_{s_{n}=1}^{k}\alpha \left(x_{n},s_{n}\right)\beta \left(x_{n},s_{n}\right), \end{array}$$

(16) $$\begin{array}{ccc} & & p\left(x_{n-1},x_{n}|s_{N}\leq k,y\right)\propto \sum_{s_{n-1},s_{n}=1}^{k}\alpha \left(x_{n-1},s_{n-1}\right)p\left(y_{n}|x_{n}\right)\\ & & \;\times p\left(x_{n}|x_{n-1}\right)p\left(s_{n}|s_{n-1},x_{n},x_{n-1}\right)\beta \left(x_{n},s_{n}\right), \end{array}$$

which involve summing out the auxiliary counting variables. Given these quantities from the E step, the form of M step remains the same as in unconstrained HMMs. The iteration between the above E and M steps leads to a local maximum of the likelihood $p(c_{x}\leq k,y)$. Notice that deriving EM algorithms under other constraints, apart from *c_x_* ⩽ *k*, can be done as above. For instance, if we wish to apply EM by assuming the number of segments to be exactly equal to *k*, we simply need to clamp the final counting variable *s_N_* to the value *k*.

We illustrate the practical consequences of the two learning approaches using 100 simulated sequences (randomly generated as the example from Figure 2). The number of segments had an empirical distribution in the range between 8 and 35 segments. We applied the EM algorithm prospectively (assuming three hidden states) under the *k*-segment constraints *s_N_* ⩽ *k*, *k* = 1, …, 50 to obtain a corresponding set of parameter estimates $\left\{{\hat{\theta }}^{\left(1\right)},\cdots ,{\hat{\theta }}^{\left(50\right)}\right\}$. We then obtained the *k*-segment paths conditioning on the corresponding parameters. We also performed parameter estimation using a standard unconstrained EM approach to obtain a single $\hat{\theta }$ and identified the *k*-segment paths retrospectively. Parameters were initialized identically so that the means of the Gaussian emission densities were spread uniformly in the range [min(y)/2,max(y)/2], each variance was set to a large value while crucially the transition matrix was initialized to an informative value, such that *A_ii_* = 10/12 and *A_ij_* = 1/12 with *i* ≠ *j*, that is close to the ground-truth transition matrix that generated each data sequence (see Section 5).

**Figure 4:**
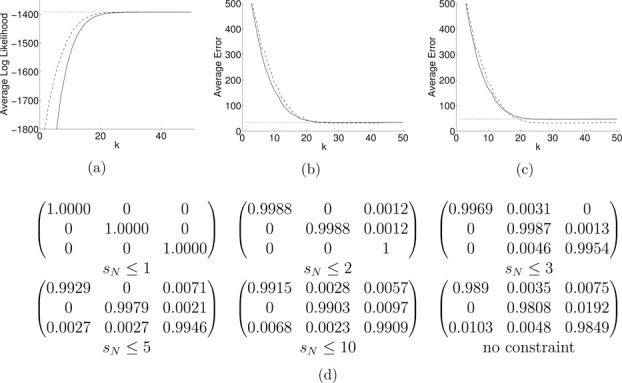
(a) Average log likelihoods for path classification for the prospective use of the constraint *s_N_* ⩽ *k* (--), the retrospective use (-), and the standard Viterbi path (..), (b) the corresponding plots for the average classification error over the hidden states assuming informative initialization of the transition matrix, and (c) the corresponding plot assuming uniform initialization of the transition matrix. (d) Examples of the estimated transition matrices under different *k*-segment constraints.

Figure 4(a) shows the average value for the log-likelihood $\log p(s_{N}\leq k,y)$ as a function of *k* for both systems. This shows that by explicitly fitting the model under an appropriate *k*-segment constraint, we achieve a higher likelihood value. In fact, by initializing the parameters in the constrained EM from the final values obtained by the standard EM should always lead to a likelihood value that is higher or equal to the corresponding value in the retrospective model. When the constraint is relaxed (as *k* increases), the likelihoods converge to the maximum value.

Furthermore, we measured the performance when doing *segmental classification*, that is, the ability to infer the underlying ground-truth hidden states that generated each sequence. Figure 4(b) shows average errors from the 100 simulations for both systems together with the average error for the standard Viterbi path of the unconstrained EM. We observe that the two approaches behave similarly and converge to the performance of standard Viterbi as *k* increases. However, if we change the initialization of the transition matrix to a less informative one, then the performance of the standard EM deteriorates while the performance of the *k*-segment EM remains unaffected, as shown in Figure 4(c). Thus, the full search in the standard EM can be more exposed to local maxima of the likelihood (associated with different estimated transition matrices that crucially affect the number of segments to be outputted) compared to the more focused search in the constrained *k*-segment EM.

The use of *k*-segment constraints during EM also provides a simple and computationally efficient mechanism to explore a wide range of different parameter estimates for the HMM. An interesting property of this is the sparsity-inducing effect that the constraint can have in the estimated values of the transition matrix. This effect is not surprising, since a bound on the number of segments essentially limits the number of transitions along the hidden path, which subsequently can result in many inferred near-zero values in the transition matrix. To demonstrate this, let us consider again the simulated sequence from Figure 2 in which we applied several times the above EM algorithm associated with the constraints *s_N_* ⩽ *k*, *k* = 1, …, 50. Figure 4(d) shows several estimated transition matrices for some of the constraints as well as the fully unconstrained case. The sparsity or shrinkage effect is clear as, for instance, when *s_N_* ⩽ 1, so that the data sequence is explained by a single segment, the estimated transition matrix becomes the identity matrix. By allowing more segments, the transition matrix gradually can have more nonzero values while when *k* is sufficiently large it becomes identical to the fully unconstrained case (in the example this occurs when *k* ⩾ 20). To conclude, it is clear that EM under *k*-segment constraints enables sparse transition matrices to be computed and this could be useful for problems involving large state spaces but where there is a priori knowledge that there may be a limited number of transitions.

Finally, when there is no prior information about which constraint to use for training the HMM, we need a mechanism to choose the best one among a set of candidates. This cannot be achieved based on the likelihood $p(c_{x}\leq k,y)$ since this quantity typically increases with *k* as the HMM becomes less constrained. Therefore, we need to resort to some external performance criterion or utility function. For instance, if in our application we care about predictive performance, as this is typical in many machine learning applications, we can rank the different models based on their generalization ability in held out test data.

### 6.2 Bayesian Approaches

It is also possible to learn an HMM under *k*-segment constraints using Bayesian inference and here we outline how this can be done using Gibbs sampling. Consider a Bayesian HMM with a prior distribution p(θ) on the parameters and a joint density

(17) $$p\left(y,x,s,\theta \right)=p\left(y|x,\theta \right)p\left(x|\theta \right)p\left(\theta \right)p\left(s_{N}\leq k,s_{\setminus N}|x\right),$$

where, as in the previous section, we assumed that the number of segments cannot exceed *k*. Notice that, while θ and s are conditionally independent given x, marginally they are dependent because of the constraint *s_N_* ⩽ *k*. We aim to compute the posterior distribution $p(x,s,\theta |s_{N}\leq k,y)$ and since this is too expensive we resort to Gibbs-type of sampling where we iteratively sample the paths (x,s) from the conditional $p(x,s|\theta ,s_{N}\leq k,y)$ and the parameters θ from p(θ|x,y). The first step corresponds precisely to the path sampling under a *k*-segment constraint using FF-BS in the augmented HMM (see supplementary materials). The second step requires simulating from the posterior conditional over parameters and clearly this will always be identical with the corresponding step when sampling in the unconstrained HMM. Also, when this step involves exact simulation from p(θ|x,y), the full algorithm is precisely Gibbs sampling, otherwise it is Metropolis-within-Gibbs where θ is sampled from a proposal distribution and then it is accepted or rejected.

In principle, the use of *k*-segment constraints can be used in an approximate Bayesian inference scheme for parallel computation of the unconstrained posterior distribution p(x,θ|y). For instance, multiple importance samplers could be simultaneously deployed to sample from the constrained densities p(x,s,θ|sN=k,y),k=1,⋯,K max , where *K*_max_ would be set to cover a reasonably large range. The constraints act as an intuitive method for partitioning the parameter space allowing the samplers to explore up to *K*_max_ regions that a standard sampler might not cover. We do not explore this aspect in detail but leave this as future work as the implementation is nontrivial as combining the samples from across the different constraints requires the conditional marginal likelihood $p(y|s_{N}=k)$, which cannot be computed by straightforward means.

## 7. EXTENDED *k*-SEGMENT INFERENCE PROBLEMS

In this section, we discuss extensions to the basic *k*-segment inference problems considered in Section 4. Specifically, in Section 7.1 we show how to solve generalized *k*-segment inference problems where we are interested in transitions of a particular type. In Section 7.2, we extend the framework in a different direction by showing how to extract highly non-Markovian events along the HMM hidden path, which consist of excursions from null states to abnormal states.

### 7.1 Counting Segments Satisfying Certain Constraints

In several applications of HMMs, we may wish to solve more general *k*-segment inference problems associated with probability events involving certain types of segments and transitions. For example, we could have a natural subgroup of states A⊂{1,...,M} and we would like to classify the observed sequence in terms of the occurrence or not of A based on the computation of the associated posterior probability. This problem consists of an example of generalized *k*-segment inference and in this section we show how this and related problems can be solved using auxiliary counting variables.

In a hidden path of an HMM (assuming an irreducible transition matrix), we can encounter *M*(*M* − 1) possible transitions that can be represented by an *M* × *M* binary matrix *C* having ones everywhere and zeros in the diagonal, that is, *C*(*i*, *j*) = *I*(*i* ≠ *j*). Such a matrix characterizes the standard *k*-segment inference problems described earlier where all segments are of interest and are all counted. When we care about a subset of transitions, we can modify *C* so that *C*(*i*, *j*) = 1, if both *i* ≠ *j* and the transition *i* → *j* belong to this subset. One way to visualize this is to think of coloring certain transitions in the HMM. Then, we will be interested in counting segments generated from only those colored transitions. Furthermore, to be flexible about the inclusion of the initial segment (which is not the result of a transition) in the probability event, we can define an *M*-dimensional binary vector μ indicating the subset of values of the initial state *x*_1_ that are of interest. Then analogously to Equation (3), we can define

(18) $$c_{x}=\mu \left(x_{1}\right)+\sum_{n=2}^{N}C\left(x_{n-1},x_{n}\right),$$

which denotes the number of segments along the hidden path x, which are compatible with the constraints (μ,C). Subsequently, we can define probability events of the form *c_x_* = *k*, *k*_1_ ⩽ *c_x_* ⩽ *k*_2_, the special events *c_x_* > *k*, etc., and subsequently formulate all associated *k*-segment inference problems as described in Section 4.1.

To solve all these new problems, we introduce again auxiliary counting variables s and define a suitable counting Markov chain p(s|x) that generates deterministically the variables in s given the path x. This chain has the same structure with Equation (7) but with the following modified conditionals:

(19) $$\begin{array}{ccc} & & p\left(s_{1}|x_{1}\right)=\mu \left(x_{1}\right)\delta_{s_{1},1}+\left(1-\mu \left(x_{1}\right)\right)\delta_{s_{1},0}, \end{array}$$

(20) $$\begin{array}{ccc} & & p\left(s_{n}|s_{n-1},x_{n-1},x_{n}\right)=C\left(x_{n-1},x_{n}\right)\delta_{s_{n},s_{n-1}+1}\\ & & +\left(1-C\left(x_{n-1},x_{n}\right)\right)\delta_{s_{n},s_{n-1}}. \end{array}$$

Here, *s*_1_ is set to one only for the subset of values of *x*_1_ compatible with μ, otherwise it remains zero and the associated initial segments are not counted. The case of counting always the first segment corresponds to the special case where μ(*x*_1_ = *i*) = 1, for each *i*, in which case *p*(*s*_1_|*x*_1_) simplifies to $\delta_{s_{1},1}$. Similarly, the conditional *p*(*s_n_*|*s*_*n* − 1_, *x*_*n* − 1_, *x_n_*) is such that *s_n_* increases only when *C*(*x*_*n* − 1_, *x_n_*) = 1 so that new segments for which *x*_*n* − 1_ ≠ *x_n_* and *C*(*x*_*n* − 1_, *x_n_*) = 0 are not counted. Clearly, counting any segment is obtained as a special case for which *C*(*x*_*n* − 1_, *x_n_*) = *I*(*x*_*n* − 1_ ≠ *x_n_*). Also, all dynamic programing recursions presented in supplementary materials are applicable to the above generalized *k*-segment inference problems by simply replacing all conditionals from the initial counting chain with the ones from the generalized counting chain defined above. Because these generalized chains can start from zero, the time complexity of all algorithms is now *O*((*k*_max_ + 1)*M* ^2^ *N*).

Finally, to illustrate optimal decoding in a generalized *k*-segment setting, we consider again the simulated data of Figure 2. Suppose, we would like to count segments from the second state only. The constraints (μ,C) we need to use are μ=[010] and *C* = [0 1 0; 0 0 0; 0 1 0] (where ; separates the rows of *C*). Figure 2(d) shows several optimal paths having 0 up to 8 segments associated with counting the second state in the HMM.

### 7.2 Extracting Excursions Using Two Layers of Auxiliary Variables

In certain applications of HMMs, such as copy number calling applications in genomics, there are often a subset of states (in the simplest case just a single state) considered as normal or null states while the remaining ones represent abnormalities. In such applications, the practitioner might be interested to identify *excursions* where the hidden path moves from any null state to abnormal states and returns back to a null state. Extracting such events using a *k*-segment formulation is challenging because an excursion has a high-order Markov structure and therefore it cannot be identified by just comparing two consecutive states. To this end, next we describe a generalization of our augmentation framework with counting variables that efficiently solves the excursion problem.

We first give a precise definition of an excursion. Suppose in HMM the states are divided into two groups: the null set N⊂{1,...,M} and the abnormal set $\bar{N}=\left\{1,...,M\right\}\setminus N$. An excursion is any subpath (*x_i_*, *x*_*i* + 1_, …, *x*_*j* − 1_, *x_j_*), with *j* − *i* > 1, where $x_{i},x_{j}\in N$ and the intermediate hidden variables (*x*_*i* + 1_, …, *x*_*j* − 1_) take values from the abnormal set. In other words, an excursion is the subpath having the start and end states clamped to normal states and with all intermediate variables clamped to abnormal values. Further, a special case of an excursion is a *restricted excursion* where the intermediate subpath (*x*_*i* + 1_, …, *x*_*j* − 1_) is clamped to the same abnormal state.

To count excursions, we introduce a new sequence of auxiliary variables $e=(e_{1},...,e_{N}),$ which signify the different phases of the excursion cycle. These variables unfold sequentially given the path x according to the following deterministic chain. Initially, *e*_1_ is set to zero so that $p\left(e_{1}|x_{1}\right)=\delta_{e_{1},0}$ and then any subsequent *e_n_* is drawn according to

(21) $$p\left(e_{n}|e_{n-1},x_{n-1},x_{n}\right)=\left\{\begin{array}{cc} \delta_{e_{n},1} & x_{n-1}\in N\;\&\;x_{n}\in \bar{N},\\ \delta_{e_{n},0} & x_{n-1}\in \bar{N}\;\&\;x_{n}\in N,\\ \delta_{e_{n},e_{n-1}} & \text{otherwise}. \end{array}\right.$$

Here, the first part of the conditional signals a new excursion where *e_n_* is set to one once a transition from a normal state to an abnormal state occurs. The second part signifies the end of the excursion where we return to a normal state. The third part replicates the previous value and deals simultaneously with both intermediate variables in the excursion subpath, in which case *e_n_* = *e*_*n* − 1_ = 1, and situations where x has started in an abnormal state and an initiation of an excursion has not occurred so far, in which case *e_n_* = *e*_*n* − 1_ = 0. The key now to count excursions is to increment a counter any time there is transition from one to zero in the path e signifying the completion of an excursion. This is achieved using counting variables s generated given e, so that *s*_1_ = 0 and any subsequent *s_n_* is drawn from

(22) $$\begin{array}{ccc} & & p\left(s_{n}|s_{n-1},e_{n},e_{n-1}\right)=I\left(e_{n-1}=1\;\&\;e_{n}=0\right)\delta_{s_{n},s_{n-1}+1}\\ & & \;+\left(1-I(e_{n-1}=1\;\&\;e_{n}=0)\right)\delta_{s_{n},s_{n-1}}. \end{array}$$

The initial HMM is augmented hierarchically with the above two layers of auxiliary variables so that

(23) $$\begin{array}{c} p(y,x,e,s)=p(y|x)p(x)p(e|x)p(s|e) \end{array}$$

is the joint density of the extended state-space HMM and each triple (*x_n_*, *e_n_*, *s_n_*) consists of the new extended hidden state. Then, by working analogously as before we can derive recursions for all types of *k*-segment inference problems associated with counting excursions. Since each variable *e_n_* takes two possible values and *s_n_* takes *k*_max_ + 1 possible values, the complexity of all dynamic programing algorithms will be *O*(2(*k*_max_ + 1)*M* ^2^ *N*), which is twice as slow as generalized *k*-segment inference.

Dealing with restricted excursions requires only a modification of the third “otherwise” part in Equation (21). In particular, this part must now be modified so that once an excursion cycle has previously been initiated, that is, *e*_*n* − 1_ = 1, we will count any transition happening between abnormal states. More precisely, this part becomes

(24) $$\begin{array}{ccc} & & p\left(e_{n}|e_{n-1},x_{n-1},x_{n}\right)=I\left(e_{n-1}=1\;\&\;x_{n-1}\neq x_{n}\right)\delta_{e_{n},e_{n-1}+1}\\ & & \;+\left(1-I(e_{n-1}=1\;\&\;x_{n-1}\neq x_{n})\right)\delta_{e_{n},e_{n-1}}. \end{array}$$

Then, the problem of counting restricted excursions is solved by constraining all *e_n_* variables to take only the two values {0, 1}, so that once an excursion cycle is been initiated we cannot transit to a different abnormal state. The time complexity of the dynamic programming recursions remains *O*(2(*k*_max_ + 1)*M* ^2^ *N*) as in the simple excursion case.

To illustrate the concept of extracting excursions, we return to the dataset of Figure 2, where we would like to count excursions so that the first and second states comprise the null set and the remaining third state is taken as abnormal. Figure 2(e) shows several optimal paths found by counting excursions where, for clarity, only the excursion segments are displayed using black solid lines.

## 8. RELATION TO OTHER METHODS

Our method formalizes and generalizes the approach of Kohlmorgen (2003) who provided the first solution (as far as we are aware) for a specific form of the *k*-segment inference problem. Kohlmorgen (2003) recognized that an exact dynamic programming solution for the optimal decoding MAP estimation problem existed. In this article, we have placed that insightful observation by Kohlmorgen (2003) within a counting Markov chain framework and showed that the use of dynamic programming can also be used for marginalization and sampling of random variables and thus, for instance, allow the computation of marginal probabilities over subset of hidden paths using the forward recursion of the F-B algorithm and simulating samples with exactly *k* segments using the FF-BS algorithm. The use of augmentation with auxiliary variables means that our framework is easily generalizable as someone can tackle different types of inference problems by constructing suitable counting chains. For instance, in Section 7, we took this forward by introducing and solving generalized *k*-segment inference problems in HMMs simply by generalizing the structure of the counting chain.

Our counting Markov chain formulation can also be related to the auxiliary Markov processes developed by Fu and Koutras (1994). Fu and Koutras (1994) developed a “finite Markov chain imbedding” (FCMI) approach that maps the original state space on to an extended state space such that classes of states in the extended space have a one-to-one correspondence with states in the original space. The extended state space is constructed such that absorbing states correspond to patterns of interest that then allows the computation of appropriate waiting time distributions associated with those patterns. These ideas have been extended and applied more recently to compute distributions of general patterns (Aston and Martin 2007), quantify uncertainty in change points in HMMs (Aston, Peng, and Martin 2012; Nam, Aston, and Johansen 2012) and more general graphical model structures (Martin and Aston 2013). Our work here provides a complimentary approach that focuses on segmental classification and the exploration of alternate sequence segmentations that we illustrate in later example applications.

In addition, there are similarities in the way we construct counting chains with that of explicit duration HMMs (Mitchell, Harper, and Jamieson 1995; Murphy 2002; Yu 2010), which consists of a modification of the original HMM where each hidden state emits not a single observation but a sequence of observations. The number of these observations is chosen randomly from a distribution. This can be thought of as introducing duration or segment length constraints in the original HMM, so that the resulting model is a hidden semi-Markov model. From a technical point, the use of counting variables in ED-HMMs shares similarities with our methodology, however, the scope of our approach is very different. Specifically, in the retrospective use of *k*-segment constraints, the counting variables are used to obtain probabilities and hidden paths in the original standard HMM, that is, we do not alter the original HMM but instead we do exploratory inference in this model, while in the ED-HMM the counting variables define a new hidden semi-Markov model that imposes segment-length constraints in the hidden sequence. When we consider *k*-segment constraints during model fit, our methodology also implies learning a hidden non-Markov model, which, however, again differs from ED-HMMs since it imposes constraints in the total number and type of segments rather than their length.

The use of efficient dynamic programing recursions has been studied extensively in the change point estimation; see, for example, Auger and Lawrence (1989), Fearnhead (2006), Fearnhead and Liu (2007), and Frick, Munk, and Sieling (2014). Traditional change point estimation algorithms allow the computation of optimal segmentations of sequential data having one up to *k*_max_ segments in *O*(*k*_max_ *N* ^2^) time, that is, these algorithms have quadratic complexity in the length of the data sequence. Recently, Killick, Fearnhead, and Eckley (2012) developed an exact algorithm whose expected computational complexity is linear in the number of observations under mild conditions. They adopted a pruning strategy to discard candidate change points and reduced the number of computations required.

Yau and Holmes (2013) also developed a decision theoretical approach for segmentation using HMMs by defining a loss function on transitions and identifying a Viterbi-like dynamic programming algorithm to efficiently compute the hidden state sequence that minimizes the posterior expected loss. The properties of the sequence predictions are modified through specification of the loss penalties on transitions as supposed to altering the transition dynamics of the Hidden Markov model. The *k*-segment algorithms developed here can also be incorporated to produce sequence predictions that minimize the posterior expected loss criterion subject to a desired *k*-segment constraint.

## 9. EXAMPLES

Next, we demonstrate the utility of *k*-segment methods in two real-world applications. Specifically, in Section 9.1 we consider the problem of copy number identification in cancer genomic sequences, while in Section 9.2 we discuss an application to text retrieval and topic modeling.

### 9.1 Genome-Wide DNA Copy Number Profiling in Cancer

First, we consider the problem of genome-wide classification of somatic DNA copy number alterations (SCNAs) in cancer. SCNAs are an important constituent of the mutational landscape in cancer and refer to numerical copy number changes that result in extra or lost copies of parts of the genome. In cancer, these alterations lead to the loss of tumor suppressor genes or the gain of oncogenes (which restrict and promote tumorigenic activity, respectively) have been identified as being associated with cancer (Beroukhim et al. 2010). Next generation sequencing or microarray technologies have allowed cancers to be probed on a genome-wide scale for SCNAs and a number of statistical models have been developed to support the analysis of this data (Loo et al. 2010; Yau et al. 2010; Chen, Xing, and Zhang 2011; Carter et al. 2012; Yau 2013). A particularly popular class of these models has used HMMs to model microarray intensities or sequencing reads as observations of a hidden (discrete) state process that corresponds to the unobserved copy number sequence.

Specifically, a single nucleotide polymorphism (SNP) microarray dataset consists of a sequence of bivariate measurements {***y***_*i*_}^*N*^_*i* = 1_ at *N* SNP locations spread across the genome. The first dimension of the measurements is known sometimes as the *Log R Ratio* values that are intensity measurements whose magnitude is proportional to the total copy number at that particular genomic location. In human genome analysis, the Log R Ratio values are typically normalized such that values approximately equal to zero correspond to a DNA copy number of two since we typically inherit one copy of every gene from each parent. The second dimension, sometimes known as the *B allele frequency*, measures the relative contribution of one of the parental alleles to the overall signal, which can allow us to determine which parental allele is lost or gained.

In Yau et al. (2010), these data sequences are modeled using a Bayesian hierarchical model specified via the following relationships:

(25) $$\begin{array}{ccc} & & y_{i}|x_{i},m,\Sigma ,\nu ∼\mathrm{Student}\left(m_{x_{i}},\Sigma_{x_{i}},\nu \right),i=1,\cdots ,N, \end{array}$$

(26) $$\begin{array}{ccc} & & x_{i}|x_{i-1}∼\mathrm{Multinomial}\left(A_{x_{i-1}}\right), \end{array}$$

where *x_i_* ∈ {1, …, *M*} denotes the copy number state at the *i*th location, {***m***_*j*_, Σ_*j*_} denotes the expected signal measurements and noise covariance for the *j*th copy number state, and *A* is a transition matrix such that *A_j_* corresponds to the transition probabilities out of the *j*th copy number state. Note, we present only an abbreviated and simplified version of the complete model by Yau et al. (2010) here. For full details, see the original reference.

Table 1 shows an example set of copy number states. Yau et al. (2010) modeled transitions between super-states as relatively unlikely events leading to a “sticky” HMM that produces relatively few super-state segments. Dynamics within super-states are modeled via an embedded Markov chain that approximates the patterns of genotypes observed in real data. The primary scientific interest is in the switching between super-states but it is necessary to fully model the complete genotypes to achieve this.

**Table 1:** Example copy number states. Each copy number state is associated with a total copy number and genotype, which tells us the number of each parental allele (A/B). The super-state corresponds to subsets of copy number states with identical total copy number and/or loss of heterozygosity (LOH) status

Copy number state	Total copy number	LOH	Genotype	Super-state
1	0	N/A	N/A	1
2	1	0	A	2
3	1	0	B	2
4	2	0	AA	3
5	2	0	AB	3
6	2	0	BB	3
7	3	0	AAA	4
8	3	0	AAB	4
9	3	0	ABB	4
10	3	0	BBB	4
11	2	1	AA	5
12	2	1	BB	5

**Figure 5:**
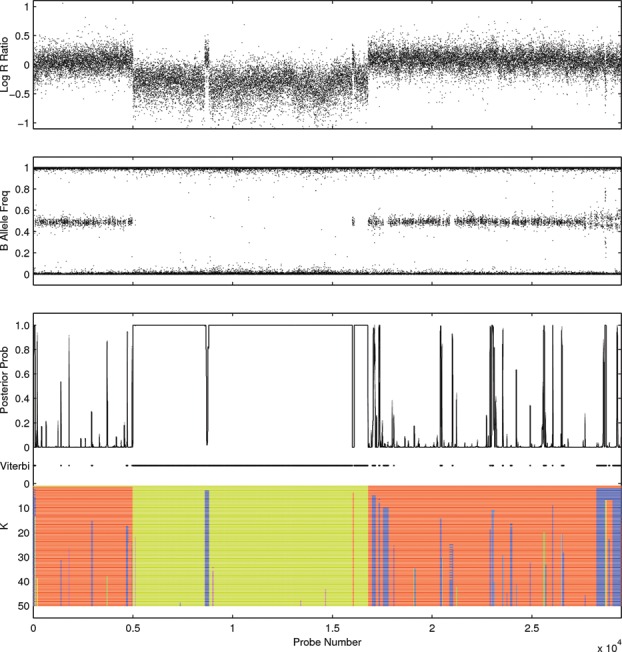
Copy number analysis of the colorectal cancer cell line SW837 (Chromosome 1) using site-wise marginal posterior probabilities of a copy number aberration from the F-B algorithm, the Viterbi algorithm (black lines indicate detected regions of aberrant copy number), and *k*-segment analysis for different fixed super-state segment numbers. Segmentation using low values of *k* provides a broad classification of the data involving large genomic aberrations, while larger values of *k* produce more detailed segmentations that may correspond to small gene deletions or amplifications.

Full Bayesian posterior inference for this type of model is prohibited by the size of the datasets (*O*(*N*) ≈ 10^6^). Yau et al. (2010) performed model fitting using the EM algorithm to compute MAP parameter estimates and condition on these to obtain MAP segmentations using the Viterbi algorithm. The F-B algorithm can also be applied to obtain site-wise posterior probabilities of state occupation. Figure 5 shows an example copy number analysis of chromosome 1 of a colorectal cancer cell line SW837 from an SNP microarray dataset using the OncoSNP software from Yau et al. (2010). The chromosome exhibits a number of copy number alterations leading to changes in the pattern of the Log R Ratio and B Allele Frequency along the chromosome. Genomic regions with nonnormal total copy number (2) can be identified from the Viterbi segmentations and the site-wise posterior probabilities.

The application of our *k*-segment methods can be used to augment these standard analyses with additional exploratory information. Figure 5 shows segmentations conditional on different fixed super-state segment numbers obtained using *k*-segments. Here, we have used the ability to count certain transitions in *k*-segment inference (based on generalized counting from Section 7.1) to good effect counting only transitions between super-states and excluding uninteresting transitions between copy number states within super-states. This means the *k*th segmentation represents the most probable copy number segmentation that involves *k* different super-state segments as supposed to *k* segments defined on the original state space, which would include transitions between states within super-states. These segmentations allow the exploration of alternative segmentation that differ from the MAP solution and yet retain segmental constraints that cannot be observed from the site-wise marginal probabilities. In this example, *k*-segments provides a coarse-to-fine representation of the genomic copy number profile for the cancer cell line allowing the investigator to choose the necessary level of detail required to answer their particular question of interest.

**Figure 6:**
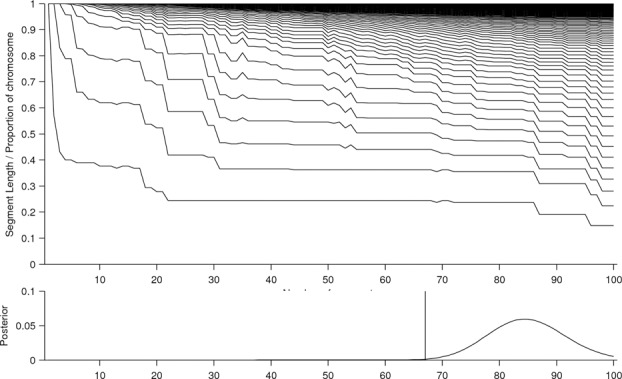
Size-ordered distribution of segment lengths found in the segmentation of chromosome 1 (top) for a range of segment numbers *k*. The posterior probability $p(c_{x}=k|y)$ (bottom) is shown alongside the Viterbi (vertical line) estimate.

Figure 6 shows that sampling from the posterior in this case would not be sufficient for obtaining a full range of qualitatively diverse sequences (as the posterior mass is mostly concentrated in the range 65–100 segments). Using the *k*-segment forward algorithm, we were able to calculate the posterior distribution exactly over the number of segments and compare this with the Viterbi solution, which involves 67 segments. Yet it is clear that the signal would be well represented with far fewer segments as the more complex segmentations simply involve large numbers of short aberrations (many of which maybe false discoveries induced by localized signal fluctuations). The potential disparity between the posterior probabilities and the potential user interpretation arises because of a model misspecification. The Markov model is only an approximation of the true (unknown) generative process and has limited expressive power. As a consequence, the sequence probabilities are not well calibrated and this effect is further exaggerated when summing over the large number of possible sequences.

### 9.2 Application to Text Retrieval Using Hidden Markov Topic Models

Next, we apply *k*-segment inference to an information retrieval task where the objective is to process long documents and extract segments referring to certain topics. For this purpose, we define a hidden Markov topic model, as those proposed in Gruber, Weiss, and Rosen-Zvi (2007) and Andrews and Vigliocco (2010), which builds upon popular topic models, such as probabilistic latent semantic indexing (Hofmann 2001) and latent Dirichlet allocation (Blei, Ng, and Jordan 2003), by assuming that the latent topics of words in ordered text follows a Markov chain.

**Figure 7:**
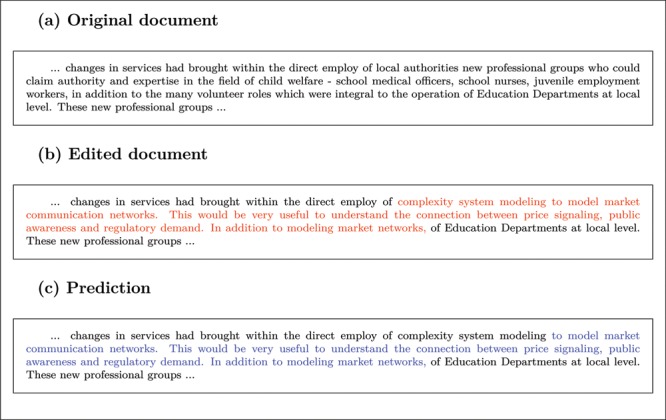
An example of detection of a text segment from the relevant topic of Economics: (a) The original test document, (b) the edited document after having randomly inserted (and replaced the original piece of text) a segment from the topic of Economics, which is shown in red, and (c) the segment predicted as belonging to the relevant topic shown in blue color. In this case, the predicted segment was classified as a correct detection since it overlaps more than 80% with the ground-truth segment shown in (b).

Assume an unknown-content (test) document *d*, which, as before, is represented by a set of words $y_{d}=\left(y_{d,1},\cdots ,y_{d,N_{d}}\right)$ that are ordered according to their appearance in the text and assumed to have been generated from an HMM. Specifically, we assume there is a path $x_{d}=\left(x_{d,1},\cdots ,x_{d,N_{d}}\right)$ such that each *x*_*d*, *n*_ ∈ {1, …, *M*} indicates the hidden topic of word *y*_*d*, *n*_. Further, the set of these topics is divided into the *relevant topics* and the *irrelevant topics* with the relevant topics being the ones from which we wish to extract text segments, estimate posterior probabilities of appearance, etc., while the irrelevant topics are unknown and document-specific topics of no interest to us. Without loss of generality, and to simplify our presentation, we shall assume *M* = 2 so that there is one relevant and one irrelevant topic. The relevant topic is described by multinomial parameters φ_*r*_ = (φ_*r*, 1_, …, φ_*r*, *V*_) so that the emission distribution that generates a word *y*_*d*, *n*_ is such that

(27) $$\begin{array}{c} p\left(y_{d,n}|x_{d,n}=1\right)=\varphi_{r,y_{d,n}}. \end{array}$$

φ_*r*_is assumed to have been estimated by supervised learning using fully labeled documents according to the equations:

(28) $$\begin{array}{c} \varphi_{r,v}=\frac{n_{v}+1}{n+V},\;\;v=1,...,V, \end{array}$$

where *n_v_* is the number of times the *v*th word appears in the labeled data and *n* is the total number of words in these data. Notice that the above is simply the Bayesian mean estimate under a uniform Dirichlet prior over φ_*r*_. Similarly, the emission distribution for the irrelevant topic, that is, *p*(*y*_*d*, *n*_|*x*_*d*, *n*_ = 2), is described by the parameter vector φ_*d*_ = (φ_*d*, 1_, …, φ_*d*, *V*_) which is a document-specific parameter to be estimated. Furthermore, the prior distribution $\pi_{d}$ and transition matrix *A_d_* of the HMM are also document-specific parameters and the full set $\left(\varphi_{d},\pi_{d},A_{d}\right)$ can be estimated via the EM algorithm while φ_*r*_ is kept fixed. In practice, we also place a conjugate Dirichlet prior over all unknown parameters so that EM finds MAP point estimates similar to those of Equation (28).

**Figure 8:**
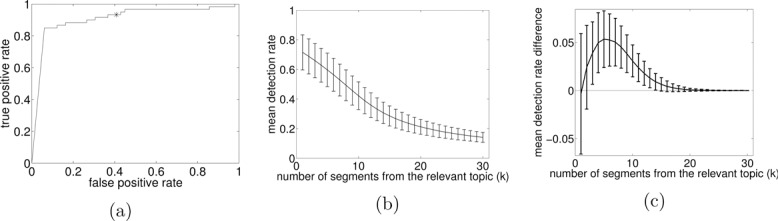
(a) Receiver operating characteristic for (-) the *k*-segment method (using $p(c_{x}>0|y)$) and (*) Viterbi, (b) mean detection rates for top-*k* systems (95% CI), (c) mean differences in detection rates of the *k*-segment method and Viterbi together (95% CI).

In the remainder of this section, we demonstrate the above system using a freely available text corpus taken from the University of Oxford electronic library. (See http://www.bodleian.ox.ac.uk/ora.) Specifically, we collected a set of 119 doctoral theses on several subjects such as History, Social Sciences, Philosophy, Law, Politics, Literature, and Economics. The topic of Economics was considered to be the relevant topic while all remaining topics were taken as irrelevant. Ten out of 119 documents were classified (according to the library database system) to be about Economics while the remaining 109 theses were scattered across the other topics. Each *d*th document was represented by a sequence of words from a dictionary of size *V* = 1260, which was defined separately by choosing all different words from a large set of freely accessible Wikipedia articles. (Following also the standard practice in topic modeling to exclude from the vocabulary very common words, of nonsemantic meaning, such as “the,” “of,” “and,” etc.) The multinomial parameters for the relevant topic of Economics was obtained by supervised learning using counts of words obtained from a small set of Wikipedia entries such as the entries Economics, Finance and Investment. Having preprocessed each document as above, we then considered two types of prediction tasks: (i) classification and (ii) detection that we describe next in turn.

*Classification.* For the classification task the objective was to predict in a test document the presence or absence of at least one occurrence of a segment from the topic Economics. The test documents consisted of the 109 theses, originally annotated as non-Economics documents, that were randomly perturbed to create a ground-truth dataset of known classification. Further simulation details are explained in supplementary materials.

Given this test dataset, the objective was to construct a binary classification system and classify each of the documents as relevant, that is, as containing at least one text segment about Economics, or as irrelevant. Each test document was processed separately by applying the EM algorithm discussed earlier. Then, to achieve probabilistic classification, the posterior probability for the occurrence of at least one segment from the relevant topic is required. It can be obtained by applying *k*-segment inference using a counting variable *c_x_* that increments only when a segment from the relevant topic occurs. Notice that this requires the use of generalized counting, as described in Section 7.1, which uses certain values for the constraints μ and *C*. (Assuming that the first hidden state in the HMM corresponds to the relevant topic and the second one to the irrelevant topic, μ=[10] and *C* = [0 0; 1 0].) Then, the posterior probability $p(c_{x}>0|y_{d})$ is computed using the forward pass of the F-B algorithm in the augmented HMM, which subsequently provides a probabilistic classifier. Using different thresholds in the classification probability, we can obtain different decision systems of varying false positive and true positive rates as shown by the receiver operator characteristic (ROC) curve in Figure 8(a). In contrast, if we were about to perform classification using the Viterbi MAP path, we can only obtain a single decision system that classifies documents as relevant or irrelevant based upon whether a segment from the relevant topic occurs or not in the Viterbi path. Such system gives a single value for the true positive and false positive rate as shown in Figure 8(a). Clearly, *k*-segment’s ability to compute nontrivial posterior probabilities allows for more flexible uses of HMMs when building decision-making systems.

*Detection.* We now turn into the second task that is concerned with the detection of individual segments within a document that belong to the relevant topic. We adopt a standard information retrieval setup that is referred to as top-*k* retrieval (Büttcher, Clarke, and Cormack 2010). This is the task of retrieving *k* patterns (typically full documents) that are most relevant to a given query among a large set of other possible patterns. Our specific top-*k* retrieval task will be to extract top-*k* text segments within the same large document and to achieve that we shall use the hidden Markov topic model. Also, to account for documents that may contain fewer than *k* segments from the relevant topic, we will relax the constraint to retrieve exactly *k* segments to the softer constraint of retrieving at most *k* segments. It is worth noticing that there is a similarity of *k*-segment problems in HMMs and top-*k* retrieval since both involve inference under counting constraints. More precisely, *k*-segments can naturally tackle the previous top-*k* retrieval task by applying optimal decoding, under the constraint *c_x_* ⩽ *k*, which finds the optimal hidden path containing at most *k* text segments associated with the relevant topic. Next, to evaluate such system in test documents with known ground-truth segments, we randomly perturbed the 109 test documents (see supplementary materials for simulation details).

To measure performance, we make use of a popular evaluation measure used in visual object detection literature. More precisely, detecting segments of certain topics in documents is similar to detecting instances of object categories in natural images. There, the detection problem is to predict a bounding box that locates an instance of an object category within the image. The well-established evaluation measure, used in the PASCAL visual object recognition challenge (Everingham et al. 2010), is the overlap area ratio. Adopting this in our case, we have that for a predicted segment *S_p_* = [*i_l_*, *i_r_*], where *i_l_* and *i_r_* are the segment start and end locations, the overlap ratio is defined by

(29) $$\begin{array}{c} r=\frac{|S_{p}\cap S_{gt}|}{|S_{p}\cup S_{gt}|}. \end{array}$$

Here, *S_gt_* is the ground-truth segment, *S_p_*∩*S_gt_* is the intersection of the predicted and the ground segments and *S_p_*∪*S_gt_* is their union. Clearly, *r* ∈ [0, 1] and values close to zero indicate poor detection while values close to one indicate strong detection. We consider as correct detections all cases when *r* exceeds the threshold of 80%; for an illustrative example of a correct detection see Figure 7. Also, to get a total document-specific performance that is normalized with respect to *k*, we average according to

(30) $$\begin{array}{c} \text{per}\;\text{document}\;\text{detection}\;\text{rate}=\frac{1}{k}\sum_{i=1}^{k_{p}}I\left(r_{i}>0.8\right), \end{array}$$

where *k_p_* ⩽ *k* is the number of predicted segments. From this we can obtain a mean detection rate that gives the overall performance in the whole test dataset. Figure 8(b) shows means detection rates for several top-*k* systems of varying values of *k*. Confidence intervals were obtained by repeating the experiment 100 times, so that in each repeat a random test dataset of 109 documents was created using bootstrapping together with the standard randomization involved in the segment insertion (see supplementary materials for simulation details).

Furthermore, it is interesting to compare *k*-segments with a system constructed using the standard Viterbi MAP path in the HMM. Standard Viterbi gives a single path that will contain a priori an unknown number of segments from the relevant topic. Thus, to get top-*k* retrieval systems (for different values of *k*), we can rank all relevant-topic segments with respect to their length so that the top-1 retrieval system simply outputs the longest segment in the list, the top-2 retrieval system outputs the two longest segments and so forth. Using the same bootstrapped 100 repeats, we also evaluated the standard Viterbi system and for each repeat we recorded the difference in mean detection rates (*k*-segment rate minus the standard Viterbi rate). Figure 8(c) displays the mean of these differences together with 95% confidence intervals and for several values of *k*. Clearly, there is a certain range of *k* values where the *k*-segment method outperforms the standard Viterbi method. Moreover, as *k* increases, the *k*-segment constraint *c_x_* ⩽ *k* becomes weaker and the corresponding optimal paths converge to the standard Viterbi MAP paths, which explains the fact that the performance of the two methods becomes identical for large *k*.

To summarize, both tasks in text retrieval presented above indicate that *k*-segment inference allows for more flexible use of HMMs, which provides us with new options when building classification and decision-making systems.

## 10. DISCUSSION

HMMs can allow for highly efficient analysis of large quantities of sequence data. However, existing methods for reporting posterior summaries from HMMs such as the Viterbi MAP path and the marginal probabilities are rather blunt providing a limited number of quantities for summarizing potentially very large sequence spaces. In a Bayesian framework, posterior sampling provides a mechanism to draw a variety of sequences but we have shown that these draws tend to come from a relatively narrow range of possibilities in practice. Furthermore, in many applications, the HMM is often a model of convenience rather than the true (unknown) generative mechanism for the data. A direct consequence of the model misspecification is that sequence probabilities may not be correctly calibrated and reliance on posterior probabilities to guide the selection of sequences may not be appropriate.

We have demonstrated that in problems where there are strong prior beliefs on segment number then the use of auxiliary counting variables allows for computationally efficient enumeration of sequences under segmental constraints. The *k*-segment algorithms we developed are generic and the augmentation scheme can be applied either a posteriori to HMMs already fitted to data or a priori during model fit. In cancer genomics, *k*-segment inference can be a useful exploratory tool that can help researchers to analyze genomic sequences at different resolutions or target events of particular types, facilitating thus the process of getting novel insight into structural rearrangements in cancer genomes. For other types of applications, which appear for instance in machine learning and pattern recognition, the proposed methods can allow to build more flexible HMM-based classification and decision-making systems, as we have demonstrated using the text retrieval example.

Regarding future work, an interesting research direction is to exploit the ability of *k*-segment inference to efficiently explore the HMM posterior distribution to provide input into constructing meta statistical models. For instance, the ability to obtain alternative explanations of the same data sequence that may have high utility to the research scientist but occur with very low probability could allow the practitioner to rerank different explanations based on his expertise and subsequently provide feedback into the model that can be used for supervised retraining.

To conclude, as datasets become larger and models more complex, we expect to see increasing need for computationally efficient methods for posterior model exploration and statistical inference under constraints. In this article, we have presented one such approach that significantly expands the statistical algorithmic toolbox of HMMs.

## SUPPLEMENTARY MATERIALS

The supplementary materials contain proofs for the auxiliary variable reformulation of k-segment problems, k-segment dynamic programming recursions, and simulation details for the text retrieval example.

## Supplementary Material

Supplementary Material
