# Supplementary material for: Statistical Inference in Hidden Markov Models Using k-Segment Constraints
Source: J Am Stat Assoc. 2016 May 5;111(513):200–15. doi: 10.1080/01621459.2014.998762 (PMC4867884; doi:10.1080/01621459.2014.998762)
Supplement: Supplementary Material [file uasa_a_998762_sm3399.pdf]

Statistical Inference in Hidden Markov Models using k-segment constraints

Supplementary Materials

Michalis K. Titsias, Christopher C. Holmes and Christopher Yau

November 27, 2014

1 Proofs for the auxiliary variable reformulation of k-segment problems

Here, we provide proofs for the correctness of the reformulation of the three k-segment inference problems presented in Section 4. Firstly, we will show that $p(\boldsymbol{x}|\boldsymbol{y}, s_N = k)$, computed via the augmented HMM, is equal to $p(\boldsymbol{x}|\boldsymbol{y}, c_{\boldsymbol{x}} = k)$ given by

$$p(\boldsymbol{x}|\boldsymbol{y}, c_{\boldsymbol{x}} = k) = \frac{I(c_{\boldsymbol{x}} = k)p(\boldsymbol{y}|\boldsymbol{x})p(\boldsymbol{x})}{\sum_{\boldsymbol{x}:c_{\boldsymbol{x}}=k} p(\boldsymbol{y}|\boldsymbol{x})p(\boldsymbol{x})}. \tag{1}$$

We have that $p(\boldsymbol{x}|\boldsymbol{y}, s_N = k)$ is defined by

$$p(\boldsymbol{x}|\boldsymbol{y}, s_N = k) \propto p(\boldsymbol{y}|\boldsymbol{x})p(\boldsymbol{x}) \sum_{\boldsymbol{s}_{\setminus N}} p(\boldsymbol{s}_{\setminus N}, s_N = k|\boldsymbol{x}). \tag{2}$$

What we need to show is that $\sum_{\boldsymbol{s}_{\setminus N}} p(\boldsymbol{s}_{\setminus N}, s_N = k|\boldsymbol{x})$ is equal to the indicator function $I(c_{\boldsymbol{x}} = k)$. Since $p(\boldsymbol{s}_{\setminus N}, s_N = k|\boldsymbol{x})$ is a deterministic distribution, given that $\boldsymbol{x}$ has k segments there will be an unique $\boldsymbol{s}^*_{\setminus N}$ such that $p(\boldsymbol{s}^*_{\setminus N}, s_N = k|\boldsymbol{x}) = 1$ and zero for all remaining $\boldsymbol{s}_{\setminus N}$s. If $\boldsymbol{x}$ does not contain k segments, $p(\boldsymbol{s}_{\setminus N}, s_N = k|\boldsymbol{x}) = 0$ for any $\boldsymbol{s}_{\setminus N}$. Thus, when $\boldsymbol{x}$ has k segments $\sum_{\boldsymbol{s}_{\setminus N}} p(\boldsymbol{s}_{\setminus N}, s_N = k|\boldsymbol{x}) = 1$, otherwise $\sum_{\boldsymbol{s}_{\setminus N}} p(\boldsymbol{s}_{\setminus N}, s_N = 1|\boldsymbol{x}) = 0$. Therefore, $\sum_{\boldsymbol{s}_{\setminus N}} p(\boldsymbol{s}_{\setminus N}, s_N = 1|\boldsymbol{x}) = I(c_{\boldsymbol{x}} = k)$ for any $\boldsymbol{x}$, from which we conclude that $p(\boldsymbol{x}|\boldsymbol{y}, s_N = k)$ reduces to the definition of $p(\boldsymbol{x}|\boldsymbol{y}, c_{\boldsymbol{x}} = k)$ from eq. (1). From that, we can immediately obtain that the term that normalizes the right hand side of (2), i.e. the quantity $p(s_N = k, \boldsymbol{y})$, is equal to $p(c_{\boldsymbol{x}} = k, \boldsymbol{y})$. This completes the proof regarding the correctness of the probability computation.

Based on the above, we can also conclude that the initial optimal decoding solution $\boldsymbol{x}^*$ is the MAP of $p(\boldsymbol{x}|\boldsymbol{y}, s_N = k)$, i.e.

$$\boldsymbol{x}^* = \arg\max_{\boldsymbol{x}} \left[p(\boldsymbol{y}|\boldsymbol{x})p(\boldsymbol{x}) \sum_{\boldsymbol{s}_{\setminus N}} p(\boldsymbol{s}_{\setminus N}, s_N = k|\boldsymbol{x}) \right]. \tag{3}$$

Given now that $p(\boldsymbol{s}|\boldsymbol{x})$ is a deterministic distribution the sum operation can be replaced by a max operation so that

$$\boldsymbol{x}^* = \arg\max_{\boldsymbol{x}} \left[p(\boldsymbol{y}|\boldsymbol{x})p(\boldsymbol{x}) \max_{\boldsymbol{s}_{\setminus N}} p(\boldsymbol{s}_{\setminus N}, s_N = k|\boldsymbol{x}) \right], \tag{4}$$

or

$$(\boldsymbol{x}^*, \boldsymbol{s}^*_{\setminus N}) = \arg\max_{\boldsymbol{x}, \boldsymbol{s}_{\setminus N}} \left[p(\boldsymbol{y}|\boldsymbol{x})p(\boldsymbol{x})p(\boldsymbol{s}_{\setminus N}, s_N = k|\boldsymbol{x}) \right], \tag{5}$$

which shows that the reformulated optimal decoding problem is equivalent to the initial one.

Finally, regarding path sampling, the FF-BS in the augmented HMM gives a pair of paths $(\widetilde{\boldsymbol{x}}, \widetilde{\boldsymbol{s}}_{\setminus N})$ that jointly comprise an independent sample from $p(\boldsymbol{x}, \boldsymbol{s}_{\setminus N}|s_N = k, \boldsymbol{y})$. Thus, $\widetilde{\boldsymbol{x}}$ alone is an independent sample from $p(\boldsymbol{x}|s_N = k, \boldsymbol{y})$.

2 k-segment dynamic programming recursions

Optimal decoding. We describe the k-segment equivalent of the Viterbi algorithm for the optimal decoding problem under k-segment constraints, i.e. for obtaining the MAP of $p(\boldsymbol{x}|\boldsymbol{y}, s_N = k)$. This algorithm will be able to solve at once all such problems from $k = 1$ up to a maximum $k = k_{max}$ by applying a single forward pass for the maximum value k_{max} which requires $O(k_{max}M^2N)$ operations. Then, by applying k_{max} backtracking operations, each scaling as $O(N)$, we can obtain all k_{max} optimal segmentations overall in $O(k_{max}M^2N)$ time.

More precisely, the Viterbi algorithm applies a forward pass where recursively $p(\boldsymbol{y}|\boldsymbol{x})p(\boldsymbol{x})p(\boldsymbol{s}_{\setminus N}, s_N = k_{max}|\boldsymbol{x})$ is maximized with respect to the pair (s_{n-1}, x_{n-1}) for any value of the next pair (s_n, x_n). This can be implemented as a propagation of a message, which is a $k_{max}M$ dimensional vector, as follows. The message is initialized to

$$\gamma(x_1, s_1) = \log p(y_1|x_1) + \log p(x_1) + \log p(s_1|x_1), \tag{6}$$

which equals $\log p(y_1|x_1) + \log p(x_1)$ when $s_1 = 1$ and $-\infty$ when $s_1 > 1$. This message then is propagated

recursively according to

$$\gamma(x_n, s_n) = \log p(y_n|x_n) + \max_{x_{n-1}, s_{n-1}} \left[\gamma(x_{n-1}, s_{n-1}) + \log p(x_n|x_{n-1}) p(s_n|s_{n-1}, x_n, x_{n-1})\right]. \quad (7)$$

$$\delta(x_n, s_n) = (x^*_{n-1}, s^*_{n-1}), \quad (8)$$

where the auxiliary message $\delta(x_n, s_n)$ simply stores the pair (x_{n-1}, s_{n-1}) that gives the maximum in (7) needed later in backtracking. Naively, the n-th recursive update can be implemented in $O(k^2_{max} M^2)$ time since each s_n takes at most k_{max} values and each x_n takes M values. However, for any given configuration of (s_n, x_n) (out of the $k_{max}M$ possible), the permissible values for s_{n-1} are either $s_{n-1} = s_n$ when $x_{n-1} = x_n$ or $s_{n-1} = s_n - 1$ when $x_{n-1} \neq x_n$. For all remaining configurations, $\log p(s_n|s_{n-1}, x_n, x_{n-1}) = -\infty$, so that these configurations need not to be checked when maximizing over (s_{n-1}, x_{n-1}) for a certain pair (s_n, x_n). Thus, the maximization in (7) can be done in M operations resulting in $k_{max}M^2$ operations for the whole n-th update. Subsequently, the full forward pass requires $O(k_{max}M^2N)$ operations. Once the forward pass is completed, we have the final message $\gamma(x_N, s_N)$ (together with all auxiliary δ messages) from which we can obtain all k_{max} optimal segmentations using backtracking as follows. For $k = 1, \ldots, k_{max}$, we first compute

$$x^*_N = \arg\max_{x_N} \gamma(x_N, s_N = k). \quad (9)$$

Then, starting from $(x^*_N, s^*_N = k)$ we backtrack recursively according to $(x^*_{n-1}, s^*_{n-1}) \leftarrow \delta(x^*_n, s^*_n)$ that recovers the optimal hidden path $\boldsymbol{x}^*$ having exactly k segments. Each backtracking requires $O(N)$ simple indexing operations.

Probability computation. For the probability computation problem, we work similarly to the above Viterbi algorithm and we compute all joint densities $p(s_N = k, \boldsymbol{y})$ for $k = 1$ up to k_{max} using the forward pass of the F-B algorithm applied to the augmented HMM. This recursively sums out each pair (s_{n-1}, x_{n-1}) for any value of the next pair (s_n, x_n), essentially passing through the so-called α message. This message is a $k_{max}M$ dimensional vector taking as initial value

$$\alpha(x_1, s_1) = p(y_1|x_1)p(x_1)p(s_1|x_1), \quad (10)$$

which equals $p(y_1|x_1)p(x_1)$ when $s_1 = 1$ and 0 otherwise. Then, the message is propagated according to the standard α recursion

$$\alpha(x_n, s_n) = p(y_n|x_n) \sum_{x_{n-1}, s_{n-1}} \alpha(x_{n-1}, s_{n-1}) p(x_n|x_{n-1}) p(s_n|s_{n-1}, x_n, x_{n-1}). \quad (11)$$

This recursion scales as $O(k_{max}M^2)$ since the summation over (x_{n-1}, s_{n-1}) can be done in $O(M)$ time by taking

advantage the structure of the counting conditional $p(s_n|s_{n-1}, x_n, x_{n-1})$. As in any α recursion in a HMM, $\alpha(x_n, s_n)$ equals the density $p(x_n, s_n, y_1, \ldots, y_n)$ so that the final message is $\alpha(x_N, s_N) = p(x_N, s_N, \boldsymbol{y})$, from which we can easily obtain

$$p(s_N = k, \boldsymbol{y}) = \sum_{x_N} \alpha(x_N, s_N = k), \tag{12}$$

for $k = 1, \ldots, k_{max}$. Clearly, since the computation of a single recursion of the α message takes $O(k_{max}M^2)$ time, the above computations require overall $O(k_{max}M^2N)$ time. Given that the joint density $p(s_N = k, \boldsymbol{y})$ has been obtained, we can compute exactly the posterior probability $p(s_N = k|\boldsymbol{y})$ by dividing with the normalization constant $p(\boldsymbol{y})$ (i.e. the overall likelihood of the HMM) obtained from the standard forward pass.

Similarly to the above, we can also define the so called backwards or β message in the extended state-space HMM. More precisely, the first β message is initialized to unity (i.e. $\beta(x_N, s_N) = 1$) and subsequent β messages are recursively obtained according to

$$\beta(x_n, s_n) = \sum_{x_{n+1}, s_{n+1}} \beta(x_{n+1}, s_{n+1}) p(y_{n+1}|x_{n+1}) p(x_{n+1}|x_n) p(s_{n+1}|s_n, x_{n+1}, x_n). \tag{13}$$

Given that each s_n takes k_{max} values, the β messages are computed in overall $O(k_{max}M^2N)$ time. Such messages are useful when applying the EM algorithm for learning an HMM under k-segments constraints as discussed in Section 6.

Path sampling. We now turn into the sampling problem where we wish to draw a path from the conditional $p(\boldsymbol{x}|s_N = k, \boldsymbol{y})$. Such a path can be obtained by sampling a pair $(\boldsymbol{x}, \boldsymbol{s}_{\setminus N})$ from $p(\boldsymbol{x}, \boldsymbol{s}_{\setminus N}|s_N = k, \boldsymbol{y})$ and then discarding $\boldsymbol{s}_{\setminus N}$. We apply the FF-BS algorithm that is based on the following decomposition

$$p(\boldsymbol{x}, \boldsymbol{s}_{\setminus N}|s_N = k, \boldsymbol{y}) = p(x_N|s_N = k, \boldsymbol{y}) \prod_{n=N-1}^{1} p(x_n, s_n|x_{n+1}, s_{n+1}, y_1, \ldots, y_n), \tag{14}$$

where the index n in $\prod_{n=N-1}^{1}$ starts from $N-1$ and decrements down to one. Applying first the forward pass described above we have the final message $\alpha(x_N, s_N, \boldsymbol{y})$ from which we can sample x_N from $p(x_N|s_N = k, \boldsymbol{y}) \propto \alpha(x_N, s_N = k, \boldsymbol{y})$. Then, recursively we go backwards and each time we sample (x_n, s_n), given the already sampled value of (x_{n+1}, s_{n+1}), from

$$p(x_n, s_n|x_{n+1}, s_{n+1}, y_1, \ldots, y_n) \propto p(x_{n+1}|x_n) p(s_{n+1}|s_n, x_{n+1}, x_n) \alpha(x_n, s_n), \tag{15}$$

where the message $\alpha(x_n, s_n) = p(x_n, s_n, y_1, \ldots, y_n)$ is known from the forward pass. Each sampling step takes

$O(M)$ time (again due to the deterministic nature of the conditional $p(s_{n+1}|s_n, x_{n+1}, x_n)$) and the whole backward sampling requires $O(MN)$ time. If we wish to simultaneously sample from all conditional distributions $p(\boldsymbol{x}|s_N = k, \boldsymbol{y})$, with $k = 1, \ldots, k_{max}$, we can do this using a single forward pass that scales as $O(k_{max}M^2N)$ and k_{max} backward sampling iterations scaling as $O(k_{max}MN)$, so the overall complexity is $O(k_{max}M^2N)$.

Furthermore, very simple and straightforward modifications of the above procedures can deal with the more general constraint $k_1 \leq s_N \leq k_2$, where $1 \leq k_1 < k_2 \leq N$. For instance, if we wish to sample a path from $p(\boldsymbol{x}|k_1 \leq s_N \leq k_2, \boldsymbol{y})$, we need to first apply the forward pass for $k_{max} = k_2$ and then perform backwards sampling exactly as described above with the only difference that initially we sample (x_N, s_N) from $p(x_N, s_N|k_1 \leq s_N \leq k_2, \boldsymbol{y}) \propto \alpha(x_N, s_N, \boldsymbol{y})I(k_1 \leq s_N \leq k_2)$. Similarly, the k-segment inference problems associated with the special event $s_N > k$ can be efficiently solved in $O((k+1)M^2N)$ time by using the absorbing counting chain[1]

$$p(\boldsymbol{s}|\boldsymbol{x}) = \delta_{s_1,1} \prod_{n=2}^{N} \left[I(x_n \neq x_{n-1} \;\&\; s_{n-1} \leq k)\delta_{s_n,s_{n-1}+1} + (1 - I(x_n \neq x_{n-1} \;\&\; s_{n-1} \leq k))\, \delta_{s_n,s_{n-1}} \right], \quad (16)$$

and then applying exactly the above algorithms by clamping $s_N = k + 1$.

Finally, it is important to notice that running k-segment inference up to some k_{max} and setting $k_{max} + 1$ as the absorbing state always gives a global summary of the posterior distribution that is guaranteed to be at least as informative as the standard Viterbi MAP path. More precisely, the events $c_{\boldsymbol{x}} = 1, \ldots, c_{\boldsymbol{x}} = k_{max}$ and $c_{\boldsymbol{x}} > k_{max}$ comprise exclusive events that make up the whole set of paths for any value of k_{max}. Therefore, the probabilities $p(c_{\boldsymbol{x}} = 1|\boldsymbol{y}), \ldots, p(c_{\boldsymbol{x}} = k_{max}|\boldsymbol{y})$ and $p(c_{\boldsymbol{x}} > k_{max}|\boldsymbol{y})$, computed based on the forward pass in the augmented HMM, always sum up to one, while the set of the corresponding $k_{max} + 1$ optimal paths must include the standard Viterbi MAP path, which will be either one of the paths from 1 up to k_{max} or the path with more segments than k_{max}. We refer to the above combined sets of probabilities and optimal paths as the $k_{max} + 1$ summary of the posterior distribution.

3 Simulation of ground-truth datasets for the text retrieval example

For the classification task we created the ground-truth dataset as follows. For each test document sequence $\boldsymbol{y}_d$ we decided with probability 0.5 to insert a number of $1 + g_d$ (with $g_d \sim$ Pois(2)) segments from the subject Economics so that these segments had random lengths from $[10, 200]$ and were also placed in random locations within the sequence $\boldsymbol{y}_d$, replacing thus the original text and with the only constraint that they didn't overlap with each other. Each such set

[1]An alternative is to assume the standard counting chain along with the event $k < s_N \leq N$. However, such a solution is very inefficient as it scales as $O(M^2N^2)$ since k_{max} must be chosen to be equal to N.

of $1 + g_d$ artificially inserted segments were also randomly selected from the 10 theses in Economics by first picking a thesis and then selecting $1 + g_d$ non-overlapping segments within that thesis text sequence. The whole procedure created a new dataset of 109 documents so that a subset of them contained segments from the relevant topic and the remaining ones did not.

For the detection task we worked similarly with the classification task discussed earlier. Particularly, again we randomly perturb the 109 test documents and insert a number of $g_d \sim \text{Pois}(10)$ segments from the subject Economics in each of the them. The insertion of segments was done exactly as described above with the only difference being that now we insert segments in all documents and their number can be much larger since $g_d \sim \text{Pois}(10)$.
